# Obstructive Sleep Apnea: From Intermittent Hypoxia to Cardiovascular Complications via Blood Platelets

**DOI:** 10.3389/fneur.2018.00635

**Published:** 2018-08-03

**Authors:** Agata Gabryelska, Zuzanna M. Łukasik, Joanna S. Makowska, Piotr Białasiewicz

**Affiliations:** ^1^Department of Sleep Medicine and Metabolic Disorders, Medical University of Lodz, Lodz, Poland; ^2^Department of Rheumatology, Medical University of Lodz, Lodz, Poland

**Keywords:** osa, blood platelets, cardiovascular diseases, hypoxia, sleep apnea

## Abstract

Obstructive sleep apnea is a chronic condition characterized by recurrent episodes of apneas or hypopneas during sleep leading to intermittent hypoxemia and arousals. The prevalence of the sleep disordered breathing is estimated that almost 50% of men and 24% of women suffer from moderate to severe form of the disorder. Snoring, collapse of upper airways and intermittent hypoxia are main causes of smoldering systemic inflammation in patients suffering from obstructive sleep apnea. The systematic inflammation is considered one of the key mechanisms leading to significant cardiovascular complications. Blood platelets, formerly not even recognized as cells, are currently gaining attention as crucial players in the immune continuum. Platelet surface is endowed with receptors characteristic for cells classically belonging to the immune system, which enables them to recognize pathogens, immune complexes, and interact in a homo- and heterotypic aggregates. Platelets participate in the process of transcellular production of bioactive lipids by delivering both specific enzymes and substrate molecules. Despite their lack of nucleus, platelets synthetize proteins in a stimuli-dependent manner. Atherosclerosis and consequent cardiovascular complications result from disruption in homeostasis of both of the platelet roles: blood coagulation and inflammatory processes modulation. Platelet parameters, routinely evaluated as a part of complete blood count test, were proposed as markers of cardiovascular comorbidity in patients with obstructive sleep apnea. Platelets were found to be excessively activated in this group of patients, especially in obese subjects. Persistent activation results in enhanced spontaneous aggregability and change in cytokine production. Platelet-lymphocyte ratio was suggested as an independent marker for cardiovascular disease in obstructive sleep apnea syndrome and continuous positive air pressure therapy was found to have an impact on platelet parameters and phenotype. In this literature review we summarize the current knowledge on the subject of platelets involvement in obstructive sleep apnea syndrome and consider the possible pathways in which they contribute to cardiovascular comorbidity.

## Introduction

Obstructive sleep apnea (OSA) is a chronic condition characterized by recurrent pauses in breathing during sleep, which lead to intermittent hypoxemia (IH), hypercapnia, arousals, reductions in intrathoracic pressure, and sleep fragmentation. The severity of the disorder is assessed by number of apneas and hypopneas occurring per hour of sleep (apnea-hypopnea index, AHI), where 5 ≥ AHI < 15 is defined as mild, 15 ≥ AHI < 30 as moderate and *AHI* ≥ 30 as severe disease (1). The prevalence of the sleep disordered breathing (SDB) is estimated that almost 50% of men and 24% of women suffer from moderate to severe form of the disorder (2). The gold standard method for diagnosis of OSA is nocturnal polysomnography (PSG). Typical symptoms of OSA include excessive daytime sleepiness, unrefreshing sleep (3), cognitive impairment (4) as well as snoring. Risk factors for OSA include obesity, male gender, older age, and higher neck circumference (5). Furthermore, it has been shown, that the patients diagnosed with OSA, more often suffer from immunological diseases that are associated with systemic inflammation (6–9) as well as metabolic disorders (10, 11). For over 30 years continuous positive air pressure is method of choice for OSA treatment, as through inhibition of airways collapse it reduces AHI and recurrent hypoxia (12).

## OSA and cardiovascular diseases

OSA has been established as an independent risk factor for cardiovascular and cerebrovascular diseases (13–15). Through chronic recurrent IH and systemic inflammation, OSA contributes to cardiovascular complications such as arterial hypertension (16, 17), myocardial infraction (18, 19), and coronary artery disease (20, 21). OSA is associated with various conditions that increase the risk of cardiovascular diseases (CVD) themselves, such as atherosclerosis and hyperlipidemia (22). Usually, frequency of cardiovascular complications of OSA increase with severity of the disorder (19), while some cardiovascular OSA co-morbidities, such as hypertension, additionally show linear association with severity of hypoxia (23). It has been shown that OSA is not only an independent risk factor for developing systemic hypertension, but also is associated with increased morning diastolic blood pressure (24). Mechanisms leading to increased risk of developing cardiovascular complications in OSA are complex and intertwine with each other. Best established among them are: increased sympathetic activation, altered vascular regulation, endothelial dysfunction, arterial hypertension, oxidative stress, and chronic systemic inflammation (25). Number of studies have shown increased sympathetic activation following IH, both in animal and human models of OSA leading to hypertension (26, 27). OSA patients have increased sympathetic traffic to peripheral blood vessels and cardiac sympathetic drive (28, 29). It leads to upregulation of renin-angiotensin-aldosterone pathway and downregulation of nitric oxide synthesis (30). Furthermore, it has been shown that CPAP treatment reduces sympathetic activity as well as increases arterial baroreflex sensitivity (31, 32).

Endothelial dysfunction is frequently recognized in OSA patients, often before clinical manifestation of CVD (33). IH regulates the release of vasoactive substances. Nitric oxide, which is the most potent vasodilator is found to be decreased is OSA patients (34, 35). CPAP treatment normalizes the level of nitric oxide (36). Additionally, increased sympathetic activity in OSA has been associated with elevations of PAI-1 (plasminogen activator inhibitor 1) and with antifibrolitic activity (37). Furthermore, it has been shown in the OSA murine models that cardiovascular remodeling induced by IH can be reversed by normoxia (38). It has been also observed that IH and hypercapnia are responsible for progression of atherosclerosis that can be partially reversed through pharmacological treatment (39). Additionally, it has been found that sleep fragmentation independently from IH also contributes to endothelial dysfunction in murine model of OSA (40).

Recurrent hypoxia-reoxygenation caused by apneas promoted notable oxidative stress among OSA patients (41). This cycle is similar to ischemia-reoxygenation cardiac injury, during which increased generation of oxygen-derived free radical is observed. CPAP therapy decreases the levels of oxidative stress in OSA patients (42). Additionally, oxidative stress is involved in regulating cellular transcription trough activation of certain transcription factors. One of the transcription factors that is activated by hypoxia is HIF-1 (hypoxia inducible factor-1), which is responsible for activation of over 100 different genes (43), including vascular endothelial growth factor (VEGF) among many others (44). Kaczmarek et al. has shown increased expression of both, HIF-1α and VEGF, in skin biopsies of OSA patient following severe nocturnal hypoxemia (45). Interestingly, in experimental murine model of OSA it has been shown that short term IH can be a protective factor against further cardiovascular complications through a positive adaptation to hypoxia stimuli (46).

Local and systemic inflammation is widely present in OSA patients. Due to IH, numerous inflammation mediators, such as TNF-α and IL-6 are increased in OSA patients (47) and their levels normalize following CPAP treatment (48). However, there are discrepancies between the results of different studies focusing on inflammatory markers in OSA. McNicholas reviewed the literature regarding CRP levels in OSA, and found some inconsistence in levels and its response to CPAP treatment (49). More studies suggest heterogeneous origins of smoldering inflammation among OSA patients. Systemic inflammation has been shown to contribute to development of atherosclerosis (50), leading to CVD. As vast majority of patients suffering from OSA are obese (51), excessive amount of central adipose tissue also contributes to level of systemic inflammation among OSA patients. Detailed description of relationship between OSA and inflammation can be found in reviews that focus on this topic specifically (52).

Numerous mechanisms that participate in CVD in OSA patients intertwine with each other. Understanding molecular mechanisms leading from IH to complications associated with OSA became crucial to developing effective therapies. Platelets, known as one of the immune cells mediating atherosclerosis, are considered as a possible pathogenic link. However, knowledge about their contribution to CVD in OSA patients is limited.

## Platelets in OSA

Research on the effect of OSA on hematological parameters is focused on its impact on blood platelets. Blood platelets play central role in hemostasis and thrombosis. They are a plausible middleman between systemic inflammation and the development of cardiovascular complications. Platelet reactivity affects blood viscosity, a dynamic parameter defined as inherent resistance of blood flow which is increased in patients with OSA in the morning (53).

Platelet indices, routinely measured as a part of a complete blood count test, were investigated as possible markers of OSA severity and certain comorbidities. Upon activation, platelets lose their regular, discoid shape for an increased surface area with lamellipodia and filopodia (54). Mean platelet volume (MPV), which reflects the average size of circulating platelets, could then serve as an indicator of platelet activity (55). Many works have shown strong correlation of MPV and thromboembolic complications, as well as worse outcome of cardiovascular events (56). Higher MPV values are associated with traditional cardiovascular disease risk factors, such as diabetes mellitus and hypertension (57). It was showed that MPV increases correspondingly to OSA severity and is associated with cardiovascular disease comorbidity (58). In two studies that excluded patients with any know cardiac disease, lung disease, diabetes mellitus, chronic renal, or hepatic disease, the only correlation found referred to patients with severe OSA. In this group of patients, MPV positively correlated with AHI (59, 60). A different research group established a correlation between MPV and both AHI and desaturation index, showing that both the number of nocturnal hypoxemia periods and the level of hypoxemia contribute to platelet activation (61). A similar study confirmed these results, showing additionally a positive correlation between MPV and high-sensitivity C-reactive protein (hs-CRP), by what it referred to the inflammatory component of OSA (62). In patients who had undergone uvulopalatal flap surgery, a procedure reducing permanent irritation due to repetitive nocturnal collapse of upper airways and snoring, MPV values were markedly decreased (63). CPAP treatment led to significant reduction of median MPV values in severe OSA patients (64, 65). However, the real usefulness of MPV as a marker of platelet activation, systemic inflammation, and predictor of thromboembolic events is far from being firmly established yet. Increased MPV could be both a possible cause and consequence of thrombosis or accelerated platelet turnover. Moreover, increased MPV is not specific and is influenced by pre-analytical conditions such as method of venipuncture or the type of anticoagulant used during blood collection, genetic polymorphisms, and lifestyle factors (66).

Platelet Distribution Width (PDW) reflects the variance in the size of circulating platelets. Formation of pseudopodia, occurring upon platelet activation, affects PDW (67). PDW is a parameter less influenced by pre-analytical conditions and thus better standardized than MPV. It was showed that increased PDW correlated with AHI in OSA patients and was significantly higher in severe OSA group (68, 69). CPAP therapy resulted in decrease of PDW (70).

Alongside with platelet shape change, changes in the composition of the phospholipid bilayer of the plasma membrane occur. Exposure of unsaturated acyl chains results in acceleration of platelet-dependent activation of serum coagulation factors. Surface receptors and adhesion molecules are upregulated what enables platelet interactions with other cells. The number of circulating platelet-lymphocyte complexes is indicative of platelet activation. It was found to reflect the severity of OSA and was independently associated with concomitant presence of hypertension (69). Not only was a platelet-lymphocyte ratio correlated with OSA severity, but regardless of OSA advancement it indicated cardiovascular complications (71). Platelets are capable of stimulating neutrophils, form aggregates with them or promote formation of neutrophil-lymphocyte complexes. Both platelet-lymphocyte ratio and neutrophil-lymphocyte ratio are increased in OSA patients, compared to a control group of snoring patients. They are parameters dependent on IH and inflammation (71).

Activated platelets release stored cytokines and chemokines in a stimuli-dependent manner. Analysis of blood serum can provide comprehensive information on the state of platelet activation and suggest a possible trigger (72). Examination of OSA patients serum showed an increased level of soluble markers of platelet activations, such as P-selectin (73) and sCD40L (74). Treatment with CPAP lowered the concentration of sCD40L by almost 50% (74). Flow cytometry analysis of blood enables to detect activated platelets upon their surface markers and specify the number of circulating platelet aggregates. Greater degree of hypoxia was predictive of platelet activation (75) and higher percentage of activated platelets was characteristic for OSA patients. Increased platelet activation was recorded during sleep and decreased over time from awakening (76). These results are consistent with another study that showed profound alteration in circadian rhythm of platelet activity in OSA patients in comparison to healthy controls. Whereas for healthy controls late night hours are the period of the lowermost platelet activity, OSA patients present the exactly reverse trend (77). It is a particularly interesting finding, as for OSA patients the peak of cardiac mortality occurs at sleeping hours (78). At the same time, not all patients respond do CPAP therapy with decrease in activated platelet percentage (79, 80).

Platelet-derived microparticles (PMP) are small fragments of platelet plasma membrane shed into the circulation. PMPs are the most abundant group of microparticles present in human blood and their number increases with platelet activation. PMPs are involved in intercellular communication and hemostasis (81). As highly procoagulant, PMPs were suggested as prognostic marker of atherosclerosis (82, 83). Even in minimally symptomatic OSA patients, PMP plasma levels are elevated (84). In more severe cases of OSA, PMP plasma levels correlate with AHI (85). Conversely, withdrawal of CPAP therapy resulted in increase of PMPs (86).

Finally, experimental stimulation of platelets with physiological stimuli (ADP, epinephrine, collagen), may assess platelet reactivity. Platelets collected from OSA patients are hyperaggregable, but do not differ significantly between patients with and without recognized cardiovascular comorbidity (87). While in healthy controls *in vitro* platelet aggregability weakened overnight, it was slightly increased in OSA patients. CPAP therapy reset platelet reactivity to level resembling that found in healthy controls (88).

Nevertheless, platelets are prone to activation upon numerous factors, including increased c-reactive protein or lipoprotein concentrations in blood (55), which commonly occur in OSA patients due to either systemic inflammation or frequent comorbidities. Due to limited availability of studies excluding confounding factors which may lead to platelet activation, it is difficult to attribute alterations of platelet parameters to IH beyond doubt. Platelet indices were mostly found to correlate with the severe form of SDB, which questions their utility in early stage of disease, when CPAP treatment is not always introduced and an antiplatelet pharmacological intervention could be beneficial.

## Platelets as immune cells mediating CVD

Platelets are the second most numerous population of human blood cells. They are responsible for initiating thrombosis at sites of blood vessel damage. Recent research describes platelets as immune cells involved in pathophysiology of various morbid states (89). In physiological conditions platelets are carried by the blood flow in close contact with the endothelial cell lining, but without adhering to it. Disruption of the vessel wall results in exposure of subendothelial structures–von Willebrand factor, type II collagen, laminin, thrombospondin, fibronectin, and vitronectin–ligands to platelets surface receptors (90). Endothelial damage by factors such as constantly elevated blood glucose (91), cigarette smoke chemicals (92), disrupted lipids homeostasis (93), turbulent blood flow, or inflammatory cytokines (94) causes persistent platelet activation and hyperaggregability. The same molecular pathways that enable blood coagulation and maintain physical barriers of the organism are the mechanisms sustaining chronic inflammation and promoting atherosclerosis. Existing atherosclerotic lesions potentiate turbulent blood flow and activate circulating blood platelets. Inflammatory milieu increases platelet response to shear stress, dyslipidemia and endothelial damage (95). A vicious loop perpetuating inflammation and pathological thrombosis is created. OSA patients often suffer from comorbidities which are established direct risk factors for endothelial damage, platelet activation, and atherosclerosis. These include: hypertension, diabetes mellitus, history of tobacco usage, and clinically present chronic ischemic heart disease. OSA with its main component intermittent hypoxia lead itself to platelet activation.

**Figure 1:**
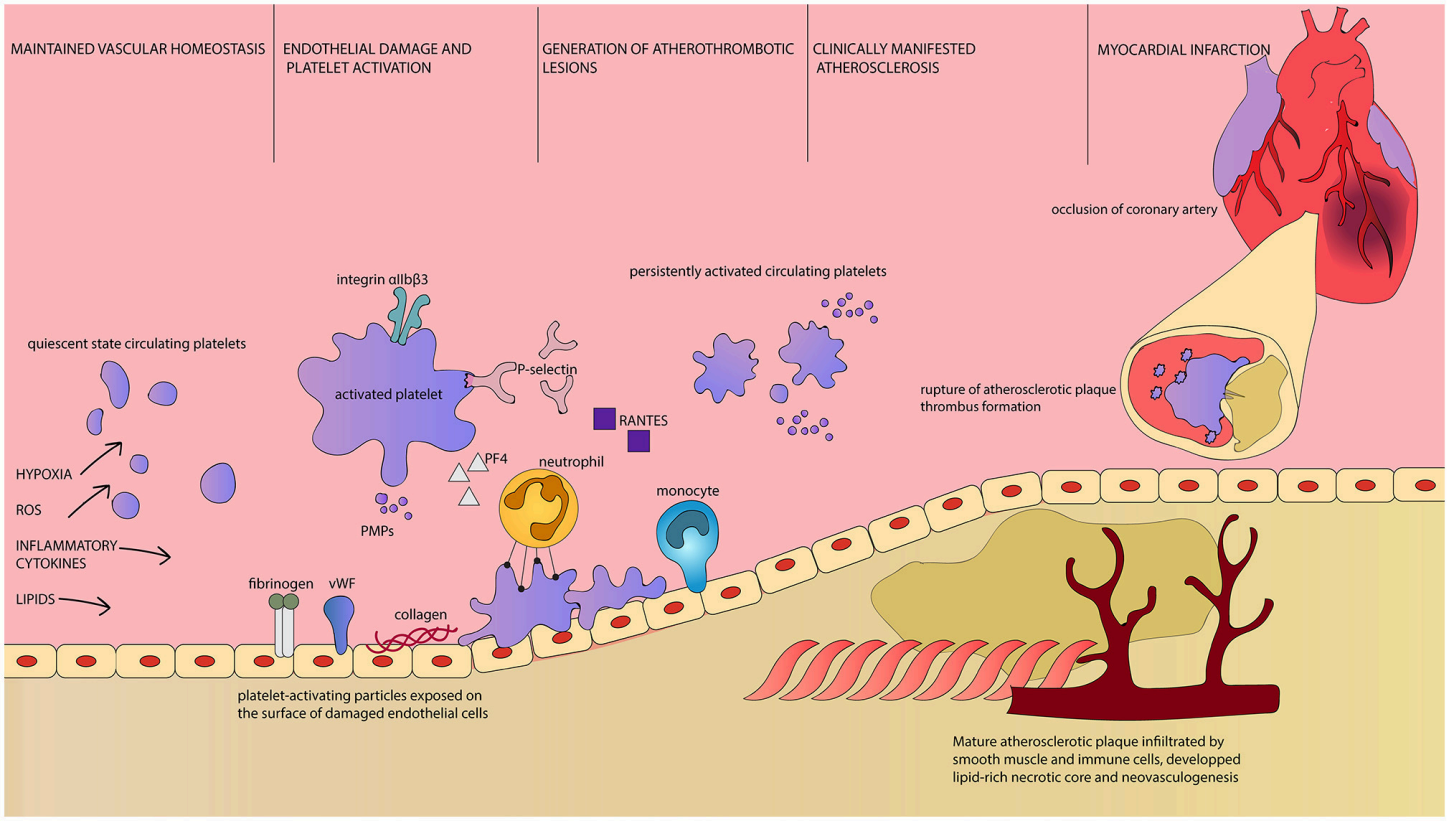
Platelets as first and final effector cells in atherogenesis and its complications. Intermittent hypoxia, oxidative stress, elevated concentration of circulating inflammatory cytokines, and lipids are factors leading to disruption of vascular homeostasis in OSA patients. Parallel to endothelial damage and demonstration of otherwise unexposed molecules, platelet activation occurs. Activated platelets are characterized by increased surface area, upregulation of surface receptors and release of PMPs as well as plethora of bioactive substances (106). Reorganization of platelet integrin αIIbβ3 conformation enables fibrinogen binding and platelet aggregation (107). P-selectin is both exposed on the surface of platelets and released. It is essential for leukocyte recruitment and enables adhesion between platelets and neutrophils, as well as platelet aggregation. Platelet-derived RANTES is deposited on endothelial cells and allows monocyte arrest and infiltration. PF4 attracts neutrophils, but also interacts with endothelial LDL receptor and promotes lipid peroxidation. PMPs are highly coagulant carriers of platelet-derived molecules, including regulatory miRNA. Platelet-neutrophil interaction is dependent on the aforementioned P-selectin and PF4 as well as CD40L and GP1βα along with their ligands expressed on the surface of neutrophil, respectively PSGL-1, CD40, Mac1 and CCL5 (89). Platelets enhance neutrophil extracellular traps formation and neutrophil oxidative burst, which can lead to tissue damage. Persistent platelet activation results in constant presence of proinflammatory and proatherogenic substances, immune cells infiltration of the endothelium and consequently, to the development of atherosclerotic plaques. Clinical manifestation of atherosclerosis indicates the presence of mature plaques with rich in lipids necrotic core, neovascularization and fragile fibrous cap. At this time, circulating platelets display prothrombotic phenotype and are easily activated. Rupture of such atherosclerotic plaque results in rapid thrombus formation. It can lead to occlusion of critical arteries, including coronary vessels, and dramatical/major consequences. ROS, Reactive Oxygen Species; vWF, von Willebrandt Factor; PMPs, Platelet-derived Microparticles; PF4, Platelet Factor 4; Integrin αIIbβ3, glycoprotein IIb/IIIa; GP1βα, glycoprotein Ib alpha chain; RANTES, regulated on activation, normal T-cell expressed and secreted, CCL5; CD40L, CD40 Ligand, CD154; PSGL-1, P-selectin glycoprotein ligand-1; CD40, Cluster of differentiation 40; Mac1, Macrophage-1 antigen, integrin αMβ2.

Activated platelets adhere to the site of vascular lesion and initiate thrombus formation. Adherent platelets release cytokines and chemokines that support inflammatory recruitment of immune cells executive in atherosclerotic plaques formations. Platelets secrete chemoattractant RANTES that binds to activated endothelium and triggers monocyte arrest, enabling the initial stages of atherogenesis (96). PMP, circulating in increased numbers during persistent platelet activation, are a source of endothelium-deposited RANTES as well (97). RANTES is the key chemokine in atherogenesis and its expression is increased in vascular wall subjected to intermittent hypoxia. Moreover, its inhibition prevents vascular remodeling induced by intermittent hypoxia (98). P-selectin, the key adhesion molecule in leukocyte recruitment, is necessary for the process of RANTES deposition (90). Soluble platelet P-selectin is a potent prothrombotic mediator stimulating fibrin deposition (99) and membrane-bound platelet P-selectin enables formation of platelet-leukocyte aggregates (100). Other cell types, including human arterial endothelial cells, also express P-selectin. The expression of P-selectin, as well as proatherogenic cytokines characteristic for endothelium, increased after stimulation with OSA patients' serum. These molecules activate platelets in a positive feedback mechanism (101). Platelet P-selectin is involved in atherosclerotic lesions formation and their further maturation and progression (100). Another platelet-derived cytokine, Platelet Factor 4 (PF4) is deposited in atherosclerotic lesions. Through interaction with low density lipoprotein (LDL) receptor PF4 interferes with LDL binding and thus promotes LDL oxidation (102). It increases binding of oxidized LDL to endothelium and contributes to fatty streaks formation within atherosclerotic lesion (103). The extent of PF4 deposition correlates with clinical symptoms of atherosclerosis and with lesion severity (104). CD40 ligand (CD40L) is expressed on the surface and secreted by activated platelets. It launches inflammatory response of the vessel wall, thrombus formation, accelerates sclerotic plaque development (105) (Figure 1). Persistent platelet activation leads to a characteristic thromboembolic end point of myocardial infarction.

Increased sympathetic activity is thought to be the main factor promoting persistent platelet activation in OSA patients. Recurrent arousals from sleep overlap with repetitive surges of sympathetic neural activity, as well as increases in concentrations of vasoconstrictive peptides and circulating catecholamines, which directly activate platelets (108). Effectiveness of CPAP therapy in reducing platelet activation confirms this interdependence (109).

Inflammatory and pro-atherogenic cytokines, circulating in elevated levels in OSA patients, may directly and indirectly activate platelets (110–112). Obesity, so tightly associated with collapse of upper airways and shift toward inflammatory phenotype, was considered to be the factor responsible for enhanced platelet activation in OSA patients (113). However, in most studies the control groups for OSA patients were age, sex- and BMI- matching subjects with excluded OSA. The level of hemoglobin deoxygenation was the variable correlating with platelet hyperreactivity (75) and CPAP therapy reduced platelet aggregability (114). At the same time increased native platelet reactivity was shown in a study of obese cohort (115). So, it seems plausible that both, OSA and obesity are independent but usually concurrent risk factors for persistent platelet activity. The issue of pathophysiological pathways in multimorbidity needs interdisciplinary research (116).

**Figure 2:**
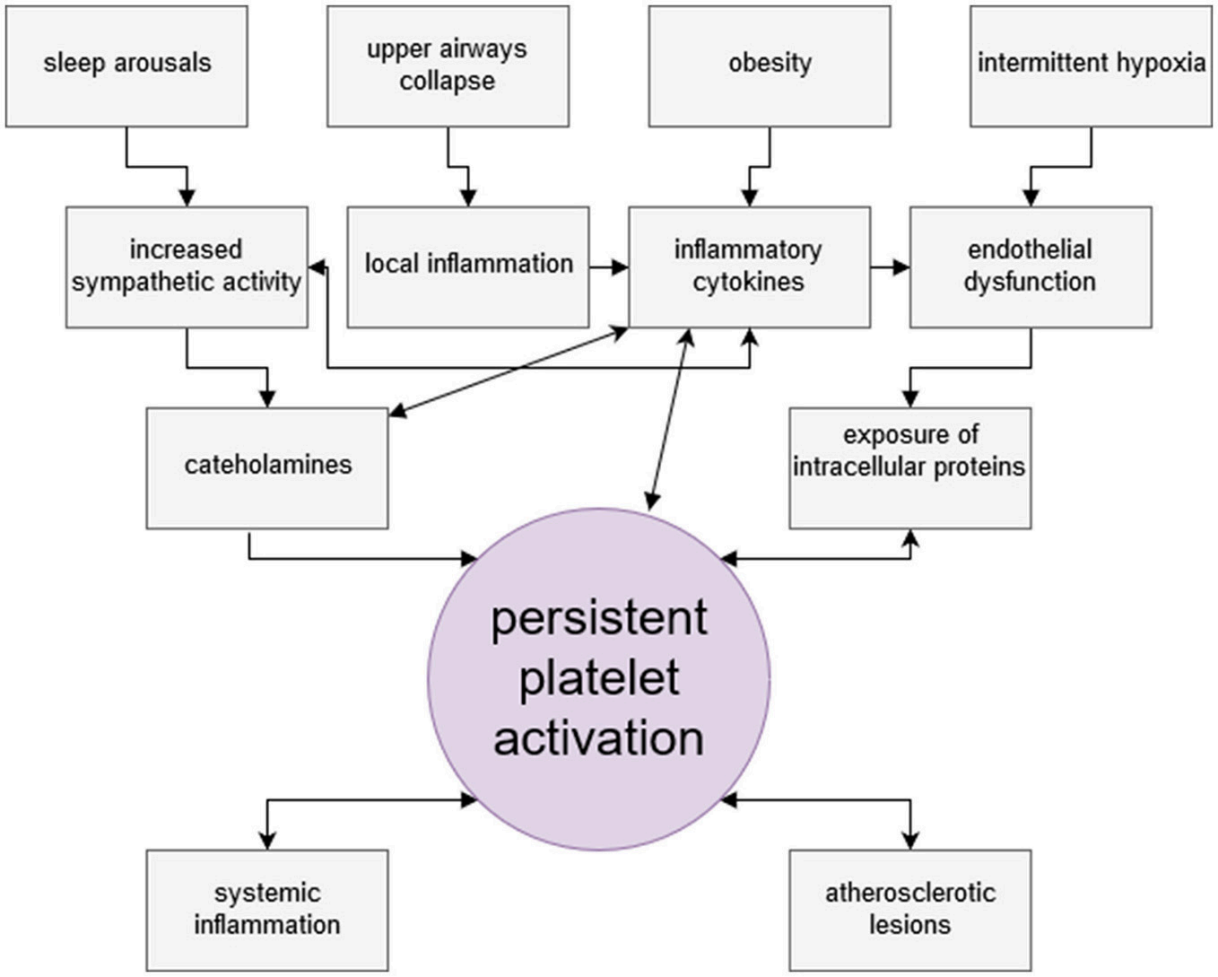
Factors leading to persistent platelet activation among obstructive sleep apnea patients. Platelets of OSA patients present thrombogenic phenotype and are hyperreactive. Among various intertwining factors leading to persistent platelet activation, four are considered as most important. Deprivation of sleep rhythm and nocturnal arousals result in increased sympathetic activity in OSA patients, manifested by elevated levels of circulating catecholamines. Noradrenaline is a potent direct platelet activator. Recurrent hypoxia events cause endothelial damage and presentation of molecules otherwise unexposed to platelets. OSA patients are characteristically obese and obesity entails the increase of the level inflammatory mediators and PAI-1 in circulation. Obesity is linked with susceptibility to upper airway collapse, which by causing mechanical irritation, contributes to sustaining inflammatory milieu. Circulating inflammatory cytokines may activate platelets, and PAI-1 inhibits antithrombotic serum activity. In turn, activated platelet secrete a plethora of bioactive, proinflammatory substances themselves. Through interaction with LDL receptor, activated platelets contribute to lipid peroxidation and, consequently, oxidative stress. Platelets are early effector cells of atherosclerosis. Persistent platelet activation may lead to serious thrombotic complications.

Finally, hypoxia itself damages endothelial lining of blood vessels and contributes to the development of endothelial dysfunction and its consequences (117). Hypoxia initiates p53-dependent pathway of endothelial cells apoptosis (118). Circulating platelets come into contact with damaged endothelium and undergo activation. Figure 2 summarizes mechanisms of increased platelet activation in OSA.

Myocardial infarction and other serious thromboembolic complications are an end point effects of persistent excessive platelet activation. OSA is a risk factor of these adverse events independently of age, BMI, hypertension, or diabetes mellitus (119). Among patients undergoing percutaneous coronary intervention, OSA was an independent predictor of lower effectiveness of antiplatelet therapy (120). Statin treatment in OSA patients lowered blood pressure and corrected lipid panel, but did not affect early atherosclerosis (121). This observation leads to questioning whether platelets should be considered a therapeutic target in OSA or rather a component of pathogenetic pathways. Studies on platelet role in OSA repetitively conclude that the gold standard treatment–CPAP therapy–affects platelet activities and reduces cardiovascular risk. CPAP also significantly improved platelet response to therapy with aspirin (122). An early manifestation of hyperreactivity of these immune cells sustaining systemic inflammation, is atherosclerosis. The presence of atherosclerotic lesions promotes platelet activation itself (95). However, platelet indices failed to predict the presence of atherosclerosis among OSA patients and were found to be a less useful marker than a non-invasive measurement of carotid intima-media thickness (123). There is paucity of studies assessing the role antiplatelet therapy in OSA patients. Current research provides evidence that in the group suffering from SDB, CPAP treatment is an intervention that effectively lowers the risk of thromboembolic events and reduces platelet activation. There is necessity of establishing guidelines of cardiovascular management in OSA patients.

## Conclusions

Available literature provides support to the fact that blood platelets in OSA patients are a viable therapeutic target to decrease CVD risk. Especially, that it has been shown that CPAP influences function of blood (87). Available anti-platelet therapies as well as other commonly administered drugs with pleiotropic effect could be beneficial to OSA patients, as treatment parallel to CPAP. Nevertheless, the molecular mechanisms behind platelet involvement in OSA and its complications still remain not fully understood. The unanswered questions comprise molecular pathways of platelet activation by hypoxia, platelet micro-RNA patterns in OSA. Since CPAP therapy adherence is limited, such studies could highlight target points of future pharmacological treatment. Additionally, more evidence for the use of platelet indices, which are routinely measured in blood count test, in monitoring OSA patients, needs to be presented to firmly establish their role in everyday clinical practice. An important issue that should be addressed by future studies is the influence of most common OSA comorbidities on platelet status and determining independent modifiers. Raising awareness of cardiovascular risk attributed to OSA among both physicians and patients is a crucial step in disease management.

## Author contributions

AG and ZMŁ created the concept of the paper, conducted literature research and wrote the manuscript. PB and JSM revised the paper.

### Conflict of interest statement

The authors declare that the research was conducted in the absence of any commercial or financial relationships that could be construed as a potential conflict of interest.

## Abbreviations

AHI: apnea–hypopnea index
BMI: body mass index
CPAP: continuous positive air pressure
CVD: cardiovascular disease
EDS: excessive daily sleepiness
HIF-1: hypoxia inducible factor-1
hs-CRP: high-sensitivity C-reactive protein
IH: intermittent hypoxemia
IL-6: interleukin 6
LDL: low density lipoprotein
MPV: mean platelet volume
OSA: obstructive sleep apnea syndrome
PAI-1: plasminogen activator inhibitor 1
PDW: platelet distribution width
PF4: Platelet Factor 4
PMP: Platelet-derived microparticles
PSG: polysomnography
SDB: sleep disordered breathing
TNF-α: tumor nectosis factor α
VEGF: vascular endothelial growth factor.
